# Role of Estrogen and Other Sex Hormones in Brain Aging. Neuroprotection and DNA Repair

**DOI:** 10.3389/fnagi.2017.00430

**Published:** 2017-12-22

**Authors:** Sandra Zárate, Tinna Stevnsner, Ricardo Gredilla

**Affiliations:** ^1^Instituto de Investigaciones Biomédicas (INBIOMED, UBA-CONICET), Facultad de Medicina, Universidad de Buenos Aires, Buenos Aires, Argentina; ^2^Departamento de Histología, Embriología, Biología Celular y Genética, Facultad de Medicina, Universidad de Buenos Aires, Buenos Aires, Argentina; ^3^Danish Center for Molecular Gerontology and Danish Aging Research Center, Department of Molecular Biology and Genetics, University of Aarhus, Aarhus, Denmark; ^4^Department of Physiology, Faculty of Medicine, Complutense University, Madrid, Spain

**Keywords:** brain aging, neuroprotection, sex hormones, estrogen, DNA repair, mitochondria

## Abstract

Aging is an inevitable biological process characterized by a progressive decline in physiological function and increased susceptibility to disease. The detrimental effects of aging are observed in all tissues, the brain being the most important one due to its main role in the homeostasis of the organism. As our knowledge about the underlying mechanisms of brain aging increases, potential approaches to preserve brain function rise significantly. Accumulating evidence suggests that loss of genomic maintenance may contribute to aging, especially in the central nervous system (CNS) owing to its low DNA repair capacity. Sex hormones, particularly estrogens, possess potent antioxidant properties and play important roles in maintaining normal reproductive and non-reproductive functions. They exert neuroprotective actions and their loss during aging and natural or surgical menopause is associated with mitochondrial dysfunction, neuroinflammation, synaptic decline, cognitive impairment and increased risk of age-related disorders. Moreover, loss of sex hormones has been suggested to promote an accelerated aging phenotype eventually leading to the development of brain hypometabolism, a feature often observed in menopausal women and prodromal Alzheimer’s disease (AD). Although data on the relation between sex hormones and DNA repair mechanisms in the brain is still limited, various investigations have linked sex hormone levels with different DNA repair enzymes. Here, we review estrogen anti-aging and neuroprotective mechanisms, which are currently an area of intense study, together with the effect they may have on the DNA repair capacity in the brain.

## Introduction

The world’s population is aging. Life expectancy at birth has increased by 6 years worldwide in the last 3 decades, and in 2050 the proportion of old adults over 60 years is estimated to reach 22% worldwide. Such an increase has a direct consequence: a rise in the incidence of age-related diseases, in particular neurodegeneration. According to different organizations like the World Health Organization (WHO), neurodegenerative disorders are, together with cardiovascular diseases, the main causes of death in western countries. More than 20% of adults aged 60 and over develop neurological disorders, dementia being the most common one. WHO estimates that around 47 million people suffer from this disorder, with nearly 10 million new cases every year. Thus, it is estimated that the total number of people with dementia will increase to near 75 million in 2030 and 132 million by 2050 worldwide. Alzheimer’s disease (AD) is the most common cause of dementia, contributing to 60%–70% of cases. Although a relationship between the development of cognitive impairment and life-style-related risk factors, such as obesity, tobacco and alcohol use has been reported, age is still the strongest risk factor for dementia and other neurodegenerative disorders (World Health Statistics, 2017).

Despite aging globally affects both male and female animals from all species, including humans, various studies have stressed that sex differences exist. Thus, females have longer life expectancies than males in mammals, and that is also the case for women vs. men in most developed countries (Promislow, 1991; Schroots et al., 1999). Since such higher life expectancy is not exclusive for humans, it cannot be attributed only to socioeconomical factors but rather reflects specific biological characteristics of both sexes. Similarly, the incidence of various age-related neurodegenerative diseases shows a sex-dependency. Thus, Parkinson’s disease (PD) has a higher prevalence and earlier onset in males compared to females (Gillies et al., 2014). Likewise, men over the age of 40 have smaller volumes in different brain areas, including the hippocampus (Coffey et al., 1998; Jack et al., 2015) and worse memory performance than age-matched women (Jack et al., 2015). On the other hand, post-menopausal women have a higher prevalence of AD than men, as well as a faster cognition decline after disease onset (Li and Singh, 2014; Zagni et al., 2016). The factors promoting the longer lifespan in women vs. men as well as sex dimorphisms in brain aging and neurodegenerative diseases are not fully understood. The positive effects of estrogen on different cellular processes, such as reactive oxygen species (ROS) production and antioxidant defense, cardiovascular protection, immune competence and telomere maintenance have been well recognized to account, at least in part, for women’s longer lifespan (Regan and Partridge, 2013; Austad and Fischer, 2016). However, it may seem paradoxical that women still live longer than men long after hormonal loss due to menopause and consequently long after having benefited from the protective effects of estrogens. Interestingly, a positive correlation between later age at menopause or longer reproductive lifespan with longevity has been recently reported (Shadyab et al., 2017). That suggests that lifetime cumulative exposure to estrogenic stimulation throughout fertile life could exert long-lasting effects and account for such sex dimorphism in longevity. This exposure would also affect brain aging and neurodegenerative processes. Altogether, the above mentioned sex differences suggest that taking sex into account as a biological variable might be critical when approaching therapies to treat neurodenegerative diseases or to delay brain aging (Young and Pfaff, 2014; May, 2016).

### Neuroprotective Effects of Sex Hormones

As mentioned above, one factor that is believed to play an important role in the sex differences observed in brain aging and neurodegeneration is sex hormone levels, in particular estrogen. It is well known that estrogen receptors (ERs) are widely distributed in the brain (Hara et al., 2015), having important regulatory function on different processes such as cognition, anxiety, body temperature, feeding and sexual behavior (Do Rego et al., 2009). The neuroprotective effect of estrogen has been stressed by several investigations. Epidemiological studies suggest that late symptom onset of PD in women may be related to such neuroprotective effect (Saunders-Pullman et al., 1999; Rocca et al., 2008; Kowal et al., 2013). It has also been suggested that the reduced concentration of sex steroid hormones after menopause may be responsible for the higher prevalence and greater severity of AD in women than men (Tang et al., 1996; Brann et al., 2007; Li and Singh, 2014). Moreover, in support of the neuroprotective effect of sex steroids, hormone replacement therapy (HRT) has been shown to have beneficial effects on different animal models (Borrás et al., 2003; Ding et al., 2013; Lu et al., 2018). Clinical data also suggest that HRT may have an effect in humans on neurodegenerative diseases such as AD (Li and Singh, 2014), PD and other age-related brain diseases like stroke (Brann et al., 2007), although concerns about its use have arisen as further discussed below.

Different animal models have been used in order to analyze how sex hormones affect neurodegenerative processes. Inhibition of aromatase, the enzyme that catalyzes the nonreversible conversion of aromatizable androgens into estrogens, results in lower estrogen levels. This inhibition is associated with altered beta amyloid deposition and more severe strokes in AD mouse models (McCullough et al., 2003; Overk et al., 2012). Similar results have been observed with aromatase knock out (KO) mice (Yue et al., 2005). Different animal models have been used for investigating the role of sex steroids in neurodegeneration and aging, being gonadectomy one of the most used. This is a standard practice in rodent hormone replacement studies, allowing investigators more control over sex hormone levels in circulation in order to study testosterone, estradiol and/or progesterone specific actions and their interaction at different levels (Frick, 2009). Moreover, ovariectomy (OVX) in female animals, especially in rodents, is a broadly used method to model women’s menopause. Like primates, rodents have reproductive cycles, which start to become irregular in middle-aged animals, i.e., 9–12 months of age in rats. However, and unlike primates, rats do not experience complete ovarian follicular loss at middle age, having chronically high circulating estradiol levels. For this reason, many laboratories often turn to the OVX rat model. Thus, using age-appropriate rats at young, middle, and old ages, surgical, peri- and natural menopause can be modeled (Morrison et al., 2006).

Several studies have shown that decreasing estrogen levels by OVX enhances neurodegenerative processes by increasing brain damage (Borrás et al., 2003; Overk et al., 2012; Yao et al., 2012; Ding et al., 2013; Kireev et al., 2014). Furthermore, restoration of estradiol levels in OVX animals by hormonal therapy reverses brain damage (Yao et al., 2012; Ding et al., 2013; Kireev et al., 2014; Lu et al., 2018). Numerous preclinical and epidemiological studies as well as some clinical trials have supported beneficial effects of HRT on memory and cognition and reduced risk for AD. Yet, an initial evaluation of results from the Women’s Health Initiative (WHI), the largest clinical trial involving postmenopausal women to date, suggested the contrary (Rettberg et al., 2014). However, the study population in the WHI considerably differed from women in previous observational studies regarding age or years after menopause onset at the time treatment was initiated. Later sub-analysis of data from the WHI trial as well as other epidemiological studies have provided evidence supporting the concept that there is a limited period of time around menopause during which HRT can exert positive effects on brain function. The “window of opportunity” theory suggests that beneficial effects of estrogens can only be achieved on a healthy brain; i.e., if HRT starts before or at the time of menopause. On the contrary, if HRT is initiated some years after menopause onset, it has detrimental effects on brain function and can even increase the risk of developing AD (Morrison et al., 2006; Brinton, 2008, 2009; Scott et al., 2012; Rettberg et al., 2014; Miller and Harman, 2017). Moreover, it has been suggested that effectiveness of HRT is likely to be dependent on additional factors, such as formulations of treatments regarding type (conjugated equine estrogen vs. 17β- estradiol, medroxyprogesterone acetate vs. progesterone), mode of delivery (oral vs. transdermal) and regimen (continuous vs. cyclic) (Miller and Harman, 2017).

Apart from being a target for sex hormones, the brain is now recognized as a steroidogenic organ, and the degree to what neurosteroidogenesis, i.e., the synthesis on neuroactive steroids in the brain, affects its function has recently started to be unraveled. Neuroactive steroids are detectable in the brain after the removal of peripheral steroidogenic organs and their levels are affected by modifications in the levels of peripheral neuroactive steroids in a region-, time after gonadectomy- and sex-manner, suggesting a compensatory mechanism in the brain to counteract the effects of peripheral hormone loss (Caruso et al., 2010; Sorwell et al., 2012; Arevalo et al., 2015). Similarly to peripheral sex steroids, neurosteroids have also been described to exert neuroprotective effects. Moreover, it has been suggested that endogenously synthesized neurosteroids may reinforce the protective effects of exogenously administered steroids (Chamniansawat and Chongthammakun, 2012; Li et al., 2013). In fact, the level of brain estrogen has been shown to determine the effect of estrogen therapy-induced protection against AD pathology in mice (Li et al., 2013). Neurosteroids are not only synthesized de novo from cholesterol. They can also be produced through transformation of blood peripheral steroid hormones into derivatives with potent neuromodulatory actions. Consequently, although neurosteroidogenesis is regulated independently of peripheral steroidogenesis, it is expected that fluctuations in plasma steroid levels affect the rate of brain steroid synthesis (Veiga et al., 2004).

Although they have received less attention than estrogens on brain function, androgens and progesterone exert neuroprotective actions as well. Testosterone and its metabolites have been described to be neuroprotective under conditions of glucose deprivation both in hippocampal neurons (Ishihara et al., 2016) and astroglial cells (Toro-Urrego et al., 2016). Similarly, testosterone has been shown to prevent dendritic atrophy in motoneurons after induced death of the surrounding neurons (Cai et al., 2017). Moreover, under chronic stress conditions, depletion of testosterone has been shown to increase susceptibility to oxidative damage in different brain areas (Son et al., 2016). Androgens have been suggested to have a positive impact on cognition (Colciago et al., 2015). This effect is likely to be mediated by testosterone and its metabolites in the hippocampus, where a high concentration of androgen receptors has been shown in hippocampal CA1 pyramidal cells (Colciago et al., 2015). Orchidectomy studies have described that testosterone replacement increases spine density in the hippocampus and at the same time improves spatial memory in rats (Jacome et al., 2016). Likewise, dihydrotestosterone (DHT) treatment also restores hippocampal spine density (Maclusky et al., 2006). Moreover, the proportion of immature dendritic spines increase after orchidectomy, suggesting that testosterone not only affects the number of synaptic spines, but also their maturation state (Li M. et al., 2012). The effects of testosterone on spine density and maturation are likely related to the brain-derived neurotrophic factor (BDNF) expression (Gao et al., 2009; Li M. et al., 2012).

Progesterone is another major sex hormone, whose best-characterized function is reproduction regulation. Progesterone receptors are broadly expressed in the brain, and they have been described to be present in all neural cell types (Brinton et al., 2008). Together with its metabolites, progesterone exerts several physiological functions in the brain. It regulates neuronal development of Purkinje cells in cerebellum (Tsutsui et al., 2011), differentiation and proliferation of oligodendrocytes (Ghoumari et al., 2005), and synaptogenesis and neuronal plasticity (Rossetti et al., 2016) among others. On the other hand, progesterone and its metabolites have been described to exert beneficial effects in various animal models of neurodegeneration and brain damage, including PD (Bourque et al., 2009) stroke (Singh and Su, 2013) traumatic brain injury and demyelination (De Nicola et al., 2009; El-Etr et al., 2015). These neuroprotective effects involve different mechanisms of action. Among them, progesterone and its metabolites activate the MAPK/ERK and PI3K/Akt pathways (Kaur et al., 2007; Baudry et al., 2013), which are well-established survival signaling pathways. In addition, progesterone also exerts neurotrophic actions regulating the expression of neurotrophins such as BDNF (Melcangi et al., 2014). Besides, progesterone modulates neuroinflammation. The anti-inflammatory effect of progesterone has been extensively investigated in an experimental multiple sclerosis murine model: autoimmune experimental encephalomyelitis (EAE). Since progesterone and its derivates promote myelin formation in the peripheral nervous system, the role that they might play in demyelinating diseases has received attention for many years. In EAE mice, progesterone itself and one of its reduced metabolites, allopregnanolone, have been shown to reduce inflammatory markers, to inhibit microglia activation and to avoid the penetration of circulating lymphocytes and macrophages in the central nervous system (CNS) (De Nicola et al., 2013, 2017; Noorbakhsh et al., 2014). This reduction in neuroinflammatory processes would mediate, at least in part, the beneficial effects of progesterone in EAE mice, which show reduced clinical severity, lower demyelination and improved neuronal function after progesterone treatment (Garay et al., 2007, 2009).

Remarkably, the effect of progesterone when delivered in combination with estrogen is not always positive. It is well known that progesterone regulates estrogen actions, particularly at the reproductive level (Graham and Clarke, 1997). At the CNS level, various studies have suggested that when both hormones are administered together, progesterone often antagonizes rather than synergizes estrogen effects (Bimonte-Nelson et al., 2004, 2006; Rosario et al., 2006; Carroll et al., 2008; Yao et al., 2011). Although the precise mechanism behind the antagonistic effect of progesterone on estrogen actions is poorly understood, some studies have suggested that it might be mediated, at least in part, by the regulation of ER expression (Pike et al., 2009).

### Sex Hormones: Mechanisms of Action in the Brain

Similar to other steroid hormones, sex steroids exert their multiple actions through binding to nuclear receptors that act as ligand-dependent transcription factors to regulate the expression of target genes (McDevitt et al., 2008). As for estrogens, two classical receptors have been described, ERα and ERβ. However, in recent years, the existence of membrane associated ER and other proteins unrelated to ERs that also trigger estrogen responses, like membrane G protein-coupled ER 1 (GPER1), has become evident (Nilsson et al., 2001; Zárate and Seilicovich, 2010; Rettberg et al., 2014). This provides an additional layer of complexity to the understanding of how sex hormones affect higher cognitive functions and other neural processes including mood, cardiovascular regulation, fine motor skills and neuroprotection (Dumitriu et al., 2010; Hara et al., 2015). Androgen and progestin receptors have also revealed both nuclear and non-nuclear forms and locations of classical and non-classical receptors (McLachlan et al., 1991; Brinton et al., 2008; Sarkey et al., 2008; Petersen et al., 2013; Li et al., 2015).

Apart from being highly expressed in regions related to reproductive behavior and neuroendocrine function like the hypothalamus, sex hormone receptors are widely distributed in the brain and are present both in nuclear and non-nuclear compartments, including mitochondria (Kelly and Levin, 2001; Boulware et al., 2007; Psarra and Sekeris, 2008; Simpkins et al., 2008; Rettberg et al., 2014; McEwen and Milner, 2017). In accordance with the complexity of the brain, sex hormones modulate not only neuronal function; they exert their actions on different cellular targets, modulating an important number of physiological processes. Thus, estrogen and progesterone have been described to regulate proliferation and maturation of oligodendrocytes (Marin-Husstege et al., 2004; Ghoumari et al., 2005) as well as local inflammation processes mediated by astrocytes and microglia (Arevalo et al., 2010). Studies in different animal models of brain injury have described that sex hormones also have an important effect in the cerebral vasculature. ERs have been localized in endothelial as well as in smooth muscle cells (Stirone et al., 2003). During stroke, estrogens exert a protective action due to theirs effect on the cerebral vasculature, increasing endothelial nitric oxide synthase activity, suppressing inflammatory markers like COX-2, and reducing leukocyte adhesion (Suzuki et al., 2009). Similarly, in a rat model of traumatic brain injury, progesterone has been described to promote angiogenic activity of endothelial progenitor cells (Yu et al., 2017).

Estrogens have an important effect on mitochondrial function as well (Klinge, 2017). Although the mechanisms by which estrogen regulates mitochondrial function are not totally understood, both direct and indirect actions have been described to contribute to such regulation. Estrogen’s beneficial effects in mitochondria are especially important in those tissues that have high demand of energy like the CNS. Along with metabolism regulation (Brinton, 2008; Klinge, 2017), estrogen exerts different actions involving mitochondrial function in neuronal tissues, including biogenesis (Kemper et al., 2014), apoptosis processes (Garcia-Segura et al., 1998; Nilsen and Diaz Brinton, 2003; Mo et al., 2013) and even morphology (Arnold et al., 2008; Hara et al., 2014). Many estrogen actions in mitochondria are mediated by the presence of ERs in these organelles, which seems to be cell-type specific. In relation to the brain, mitochondrial ERs have been suggested to be present in primary cultured rat neurons, murine hippocampal cell lines (Yang et al., 2004), neurons and glia of rat hippocampus (Milner et al., 2005; Herrick et al., 2006) and also in pre- and post-synaptic mitochondria of hippocampal neurons (Milner et al., 2008). The presence of ERs within mitochondria suggests that estrogen might modulate mitochondrial function by directly affecting transcription of mitochondrial DNA (mtDNA). In fact, mitochondrial ERβ has been described to bind to estrogen response element-like sequences in mtDNA (Demonacos et al., 1996; Chen et al., 2004). However, estrogen actions on mitochondria are not exclusively related to such mechanism. Estrogen also regulates mitochondrial functions through their classical nuclear mechanism, i.e., transcriptional regulation of nuclear-encoded mitochondrial proteins. It is known that estrogen regulates the nuclear transcription of different proteins affecting mitochondrial function such as nuclear respiratory factor-1 (NRF-1) and peroxisome proliferator-activated receptor-gamma coactivator 1 (PCG-1) (Kemper et al., 2013; Klinge, 2017). Hence, this regulation is critical for the activation of nuclear genes encoding proteins involved in mitochondrial biogenesis as well as in the mitochondrial electron transport chain complexes (Scarpulla, 2008; Klinge, 2017). It also regulates the transcription of mitochondrial transcription factor A (TFAM), which translocates into mitochondria and initiates transcription and replication of mtDNA (Virbasius and Scarpulla, 1994; Kang et al., 2007).

Effects of estrogen in mitochondria might be especially relevant in the brain since the accumulation of mtDNA mutations and the related mitochondrial dysfunction have been suggested to play a critical role in the process of brain aging and in the onset of neurological disorders (Barja, 2004; Cantuti-Castelvetri et al., 2005; Kujoth et al., 2007). Accordingly, increased levels of oxidative modifications and mutations in mtDNA occur in the brain during normal aging (Melov, 2004; Beal, 2005; Vermulst et al., 2007), with enhancement of these levels in neurodegenerative diseases such as AD and PD (Gabbita et al., 1998; Sanders et al., 2014). One of the main factors contributing to mtDNA instability, both during brain aging and in neurodegenerative diseases, is the decline in mtDNA repair capacity (Imam et al., 2006; Weissman et al., 2007a; Gredilla, 2010; Gredilla et al., 2012). Different DNA repair pathways have been described both in the nucleus and mitochondria (Gredilla et al., 2010a; Jeppesen et al., 2011). The major ones are base excision repair (BER), mismatch repair (MMR), nucleotide excision repair (NER) and double-strand break repair. The main pathway taking place in mitochondria is BER, which repairs mtDNA modifications caused by alkylation, deamination and oxidation. A brief description of these repair pathways and the effect of sex-hormones on them will be described later in this review.

## Sex Hormones and Brain Aging

Aging is an inevitable physiological process orchestrated by a plethora of molecular mechanisms that interact to alter body homeostasis, eventually leading to organismal functional decline and disease. While for many years the brain was not considered to be a sex-hormone-responsive organ, except the hypothalamus for reproductive function regulation, it is now well accepted that the entire brain is both a target and a source of sex hormones (Acaz-Fonseca et al., 2016; McEwen and Milner, 2017). Sex hormones exert numerous protective and antioxidant actions in the adult brain increasing neural function and resilience and promoting neuronal survival. As the organism age, a relatively rapid loss of ovarian hormones in the female after menopause, and a gradual but indeed significant decline of testosterone in men occur. Thus, it is not surprising that reproductive senescence both in males and females has a negative impact on neural function and represents a significant age-associated risk factor for neurodegenerative diseases, such as AD (Barron and Pike, 2012).

### Sex Hormones and Synaptic Plasticity during Aging

The key role of estrogens and ERs in the synaptic basis of cognitive functions mediated by the hippocampus and prefrontal cortex (PFC) is well recognized (Dumitriu et al., 2010; Hara et al., 2015). Both ERα and ERβ are localized in synaptic terminals and dendritic spines, dendritic shafts, axons and glial cell processes in a membrane-associated manner (Milner et al., 2005; McEwen and Milner, 2007), suggesting that estrogen mediates its effects on synapses locally rather than via regulating nuclear transcription. As the brain ages, changes in the pattern of ER expression and/or in differentially activated signaling pathways in these brain areas have been suggested to be the basis for the detrimental effects of aging in memory and learning. Briefly, synapse number and spine density decrease with natural or surgical depletion of ovarian hormones in the CA1 area of the hippocampus from female rats (Gould et al., 1990; Woolley and McEwen, 1992, 1993; Adams et al., 2001). Unlike what is observed in young animals, estrogen treatment cannot restore synapse and spine density levels in aged animals, suggesting that the hippocampus becomes unresponsive to estrogen effects with age (Adams et al., 2001). Furthermore, the finding that the number of ERα-containing synapses in the hippocampus of old female rats decreases to half the number found in young animals could explain loss of estrogen actions in the aged hippocampus, which has been suggested to be the basis for lower brain plasticity with aging (Adams et al., 2002). Unlike the hippocampus, hypothalamic levels of ERα and progestin receptor are maintained with age in female rhesus monkeys, while membrane GPER1 expression is increased, indicating that the aged hypothalamus retains the ability to express steroid hormone receptors at levels comparable to young adults (Naugle et al., 2014).

Despite being extensively homologous, ERα and ERβ diverge in their expression and action in the brain. They are widely expressed throughout the adult brain and their expression is differentially regulated in aging and by estrogen treatment. Like ERα, the levels of synaptic ERβ are reduced with age in the hippocampus of female rats. However, ERβ levels are increased following estrogen treatment both in young and old OVX rats, indicating that ERβ-mediated effects at the synaptic level are maintained during aging in the female rat hippocampus. It suggests that ERβ would be a more sensitive target to estrogen actions in the aged female brain (Waters et al., 2011). Since ERβ signaling has been associated with altered synapse formation and plasticity (Szymczak et al., 2006; Waters et al., 2009), the shift to decreased ERα/ERβ ratio has been proposed to be a major contributor to the age-induced loss of synapse formation by estrogens (Waters et al., 2011). Also, since ERα and ERβ are linked to unique second messenger pathways that can oppose one another, the altered ERα/ERβ ratio can contribute to deficits in specific signaling pathways, affecting memory and plasticity in old animals (Waters et al., 2011). Moreover, clinical studies have shown a positive correlation between Mini Mental StateExam (MMSE) score and nuclear ERα levels in the frontal cortex of AD patients, suggesting that decreased ERα responsiveness is directly associated to severity of cognitive impairment (Kelly et al., 2008). On the contrary, an age-realted increase in the ERα/ERβ ratio has been reported in cortical astrocytes from both male and female rats, which correlated with lower glial trophic support to neuronal function. The increased ratio was suggested to be associated to both long-term potentiation and spatial memory impairment (Paris et al., 2011; Arimoto et al., 2013; Yin et al., 2015).

Like females, the male brain is also responsive to variations in androgen levels at the synaptic level. The density of dendritic spines in the hippocampus has been reported to be modulated *in vivo* by androgen depletion and replacement (Leranth et al., 2003). Gonadectomy in male rats decreased CA1 spine synapse density compared to sham-operated animals (Jia et al., 2013). Since it can be metabolized into the androgen DHT and estradiol, testosterone can mediate its effects through androgen and/or estrogen pathways. Treatment of gonadectomized rats with DHT or testosterone propionate but not with estradiol restored spine synapse density to similar levels of those found in intact males, suggesting a direct role of androgens through androgen receptors rather than indirectly via local estradiol biosynthesis in hippocampal synaptic plasticity (Leranth et al., 2003). Similar results were obtained in SAMP8 mice, an animal model of accelerated aging (Jia et al., 2016; Pan et al., 2016).

### Sex Hormone and Growth Factor Interaction during Aging

A functional interplay between ERs and growth factor receptors, such as insulin-like growth factor-1 (IGF-1) or BDNF, has broadly been shown to take place in the brain. Hence, it is expected that conditions that affect the expression and/or activity of these receptors have a reciprocal negative impact on the multiple processes regulated by these systems, from the control of hormonal homeostasis and reproduction to learning and cognition. For example, estrogen-induced transport of glucose in the brain through the insulin-sensitive glucose transporter GLUT-4, adult hippocampal neurogenesis and protection against stroke are processes that require the coupling between ERα and IGF-1 receptor, providing further evidence for the interplay between these two systems in promoting enhanced neuronal metabolism and neuroprotection (Cardona-Gómez et al., 2002; Garcia-Segura et al., 2010; Arevalo et al., 2012; Sohrabji, 2015; Huffman et al., 2017). Besides, in aged OVX animals, which had undergone estrogen replacement treatment during middle age, estrogen-induced improvement in memory function was abolished by treatment with an IGF-1 receptor inhibitor. This finding indicates that estrogen may exert part of its lasting effects on the hippocampus and memory through the IGF-1 receptor signaling pathway (Witty et al., 2013). In female rats, both reproductive senescence and OVX have been shown to consistently decrease the levels of IGF-1 gene expression, which correlates with increased expression of genes involved in Aβ generation (Rettberg et al., 2014). In addition, clinical studies have shown that patients with AD have decreased expression of insulin receptors and impaired insulin signaling in brain areas susceptible to AD pathology, which could account for the early cognitive impairment seen in these patients (Schiöth et al., 2012). These studies suggest that impaired brain estrogen/ER and IGF-1/IGF-1 receptor systems may account, at least in part, for the women’s well known higher vulnerability to develop AD after menopause. Despite estrogens and androgens share metabolic pathways and functional properties, far less research has examined a functional link between IGF-1 and androgens in the brain (Huffman et al., 2017). However, some studies have shown that IGF-1/androgen interactions promote beneficial effects in neuroprotection (García-Fernández et al., 2008; Puche et al., 2008). On the other hand, BDNF is a crucial molecule for synaptic plasticity and hippocampal memory formation (Heldt et al., 2007; Bekinschtein et al., 2014). BDNF and estrogens activate a number of common signaling pathways, which converge in the induction of growth, survival, neural plasticity and learning. Estrogens can also induce BDNF gene expression through direct binding to an estrogen-sensitive response element (ERE) on the BDNF gene or by increasing neural activity that in turn upregulates BDNF (Scharfman and MacLusky, 2006). Serum BDNF levels have been reported to decline with increasing age in both men and women (Shimada et al., 2014). In addition, a significant drop in serum BDNF levels was found in women after menopause, suggesting that ovarian hormone and BDNF circulating levels are tightly associated (Bus et al., 2012). Interestingly, a recent report has shown that working memory-related hippocampal function is differentially modulated by estradiol in women carrying the specific BDNF Val^66^Met functional single-nucleotide polymorphism (SNP; Wei et al., 2017). A decrease in BDNF expression has been observed both during aging and after OVX in murine hippocampus (Singh et al., 1995; Sohrabji et al., 1995; Chapman et al., 2012; Perovic et al., 2013; Lu et al., 2014) and estrogen replacement treatment to OVX rats has been shown to increase BDNF mRNA or protein levels (Singh et al., 1995; Sohrabji et al., 1995; Gibbs, 1998; Kiss et al., 2012; Lu et al., 2014). Recently, it has also been suggested that estrogen-enhanced consolidation of multiple forms of hippocampal memory in middle-aged rats is associated with the induction of BDNF protein levels through non-classical cell signaling mechanisms involving epigenetic regulation of the BDNF gene (Fortress et al., 2014). Although there has been substantial growth of new data on the functional consequences of the interplay between sex hormones and these growth factors in recent years, many key aspects remain to be addressed. In particular, an area that warrants further study is to ascertain the role this interaction plays during aging and menopause, when the levels of sex hormones and IGF-1/BDNF decline and the cells and tissues that respond to them undergo both metabolic and functional changes (Garcia-Segura et al., 2010; Sohrabji, 2015).

### Sex Hormones and Mitochondrial Function during Aging

Despite comprising only 2% of the body’s mass, the brain consumes 20% of the body fuel to sustain its high demand of energy in the form of ATP, making it highly dependent on proper mitochondria function (Rettberg et al., 2014). As mentioned before, estrogens have beneficial effects on brain energy metabolism, increasing blood flow and glucose uptake and enhancing aerobic glycolysis coupled to the citric acid cycle, mitochondrial respiration and ATP generation (Brinton, 2008). Not surprisingly, a strong link between the drop in circulating ovarian hormones and reduced brain bioenergetics in women during menopause and in animals undergoing natural or surgical reproductive senescence has been reported (Maki and Resnick, 2000; Rasgon et al., 2005; Yao et al., 2009, 2010, 2012). In line with this, it is now recognized that normal aging and several age-related diseases, such as AD and PD, are related to mitochondrial dysfunction (Chakrabarti et al., 2011; Johri and Beal, 2012). The common features observed in aging and AD regarding mitochondria have recently been reviewed (Grimm et al., 2016a). Briefly, the aging process is characterized by decreased mitochondrial activity, including impaired oxidative phosphorylation, reduced expression and activity of respiratory chain complexes and decreased antioxidant defenses (Grimm et al., 2016a). Extensive evidence indicates that the decline in brain mitochondrial function observed in reproductive senescent female animals is caused by loss of ovarian hormones (Yao et al., 2009, 2010, 2012). Also, considering that the first steps of steroidogenesis take place in mitochondria, it is reasonable to hypothesize that age-related accumulation of mitochondrial deficits may have a detrimental effect in steroid biosynthesis and comprise a potential pathogenic mechanism leading to neurodegeneration (Velarde, 2014).

OVX in young adult animals has been shown to induce adverse effects in brain mitochondrial bioenergetics similar to those found in aged animals, including reduced respiration and ATP production rates, increased oxidative stress and decreased expression and/or activity of metabolic enzymes within this organelle (Irwin et al., 2011; Shi et al., 2011; Yao et al., 2012; Gaignard et al., 2015). A recent report has shown that OVX induces mitochondrial dysfunction in terms of reduced active respiration and ATP production likely associated to alterations in the mitochondrial membrane lipid profile in the hippocampus (Zárate et al., 2017). In particular, OVX induces changes in the fatty acid profile of mitochondrial membranes rendering them more prone to peroxidation, a feature also observed during aging in an organ-dependent manner (Pamplona, 2008). Interestingly, OVX also induces a specific decrease in cardiolipin content and changes in its fatty acid composition. Importantly, cardiolipin is an essential component of mitochondria membranes that plays a crucial role in several mitochondrial processes such as oxidative phosphorylation, apoptosis, mitochondrial protein import and supercomplex formation (Claypool and Koehler, 2012). Reduced cardiolipin content, alterations in its acyl chain composition and/or increased cardiolipin peroxidation have been linked to mitochondrial dysfunction in multiple tissues during aging and in neuropathological disorders (Monteiro-Cardoso et al., 2015). Overall, these studies suggest that loss of ovarian hormones accelerates the decline in mitochondrial bioenergetics promoting a premature aging phenotype (Yao et al., 2009). In line with this, alteration in mitochondrial membrane lipid composition, especially in cardiolipin content, could be an additional player in the aging effects of ovarian hormone loss contributing to the early bioenergetic decay during menopause (Zárate et al., 2017).

Although activation of both ERα and ERβ favors mitochondrial function, ERβ activation often results in greater mitochondria functional capacity (Irwin et al., 2012; Yao et al., 2013). It has also been suggested that estrogen effects in brain metabolism mainly relies on ERβ signaling through directly promoting mtDNA gene expression, mitochondrial antioxidant defenses and oxidative and calcium buffering capacity (Nilsen and Diaz Brinton, 2003; Yang et al., 2004; Simpkins et al., 2008; Rettberg et al., 2014).

In addition to be the main source of ATP in cells, mitochondria also play important roles in other cellular functions, such as cell growth and differentiation, regulation of intracellular calcium homeostasis, apoptosis, alteration of the cellular redox state and synaptic plasticity (Grimm et al., 2016b). Mitochondria are considered the major source of ROS production in cells under physiological conditions. Electrons leak the electron transport chain during mitochondrial respiration, combining with molecular oxygen to generate ${\text{O}}_{\text{2}}^{\text{−•}}$, which subsequently can be converted to H_2_O_2_ by superoxide dismutase (SOD). When compared to most other tissues, the brain has very low antioxidant capacity and it is subjected to particularly high levels of oxidative DNA damage. Considerable evidence supports the role of oxidative damage in the aging process (Golden et al., 2002; Samarghandian et al., 2016) and an increasing number of studies implicate ROS as an important contributor to cognitive impairment during aging as well as in age-associated neurodegenerative diseases (Grimm et al., 2016b). Estrogens have well-known antioxidant effects. Clinical evidence points to lower brain oxidative stress and better antioxidant defenses in premenopausal women compared to men, parameters which gradually decrease as women age or if they undergo bilateral oophorectomy (Mandal et al., 2012; Bellanti et al., 2013; Rekkas et al., 2014). Similar results were obtained in animals, where brain mitochondria from young adult females showed lower peroxide production and higher levels of MnSOD, glutathione (GSH) and glutathione peroxidase (GPx) compared to males of the same age (Borrás et al., 2003). Estrogens also increase the expression of peroxirodoxin 5 and glutaredoxin in brain mitochondria (Nilsen et al., 2007). Remarkably, OVX blunted the differences in peroxide and GSH levels between females and males while estrogen treatment prevented them, highlighting the protective effects of this steroid against oxidative stress (Borrás et al., 2003). A similar pattern of a positive correlation between circulating testosterone levels and the activity of antioxidant enzymes both in serum and in the hippocampus have been reported in men and in orchidectomized rats, respectively (Meydan et al., 2010; Cunningham et al., 2014). Again, these effects were prevented in animals after testosterone administration (Meydan et al., 2010).

Some brain areas seem to have a particularly high vulnerability to the effects of sex hormone deprivation, aging and oxidative stress. Thus, several studies have reported a link between hippocampal synaptic decline, cognitive impairment and increased risk of neurodegeneration after OVX in animal models and menopause in women (Morrison et al., 2006; Brinton, 2009; Velarde, 2014; Hara et al., 2015). Mitochondrial dysfunction and oxidative damage are also more evident in the hippocampus than in brain cortex or whole brain in aged male rodents (Navarro et al., 2008). Moreover, OVX severely induces a decrease in the activity of SOD together with an increase in the pro-oxidant enzyme monoamine oxidase (MAO) in the hippocampus but not in the cortex of young rats (Huang and Zhang, 2010). Overall, increasing evidence indicates that the hippocampus is an early target of aging, sex hormone loss and oxidative stress (Navarro et al., 2008; Paradies et al., 2011; Hara et al., 2015).

Another brain region highly sensitive to ovarian hormones is the PFC, an area tightly associated to cognitive function in humans. A recent report has shown that aging in non human primates induces morphological changes in mitochondria from this brain area leading to a mitochondrial phenotype related to enhanced oxidative stress and ROS production. Noteworthy, this correlates with worsening of working memory. Interestingly, OVX promoted similar effects in mitochondrial morphology and cognitive behavior, which was reversed by estradiol treatment. These studies suggest that estrogen effects on PFC-related memory can result from its antioxidant capacity leading to improved mitochondrial health (Hara et al., 2014).

### Sex Hormones and Neurosteroidogenesis during Aging

Another process that has been described to be affected by aging is the synthesis of neurosteroids. The transport of cholesterol from the outer to the inner mitochondrial membrane is the first and rate-limiting step in steroidogenesis. For many years, there has been general agreement that this trafficking relies on the activity of at least two proteins, steroidogenic acute regulatory protein (StAR) and translocator protein of 18 kDa (TSPO), previously known as peripheral benzodiazepine receptor (PBR; Veiga et al., 2004; Acaz-Fonseca et al., 2016). However, recent research using genetic depletion of TSPO both in *in vivo* and *in vitro* models has challenged the view of TSPO as a critical enzyme for steroidogenesis (Selvaraj et al., 2015). A recent report suggested that TSPO may be functionally redundant in achieving baseline steroidogenesis although it may play an important role in maintaining androgen levels during aging (Barron et al., 2017). It is important to stress that the expression and activity of both enzymes are increased with aging and after brain injury, suggesting that the production of brain steroids can be modulated as a protective mechanism to cope with decreased peripheral steroids or pathological conditions (Veiga et al., 2004).

Also, other enzymes catalyzing different steps in neurosteroidogenesis are differentially expressed in neurons and glia in a region and pathophysiological-condition manner. Under physiological conditions, neurons are the main sites for brain estrogen production, relying on their high expression of aromatase (Acaz-Fonseca et al., 2016). However, enhanced aromatase expression in astrocyes has been reported following brain injury in rats and also in the human PFC in the late stages of AD, suggesting that neuronal impairment can induce estrogen production as a glial protective mechanism against neuronal death (Veiga et al., 2004; Luchetti et al., 2011; Acaz-Fonseca et al., 2016). A recent report has shown decreased levels of aromatase in the hippocampus of aged female mice when compared to the levels detected in adult mice (Zhao et al., 2017). OVX also downregulates aromatase gene expression in the hippocampus of middle-aged rats (Sárvári et al., 2014). Female brain-derived estradiol levels have been reported to mirror estradiol circulating levels and thus significantly decline in postmenopausal compared to premenopausal women (Rosario et al., 2011). Since the level of the estradiol precursor and aromatase substrate testosterone is also decreased in the female cerebral cortex after OVX (Caruso et al., 2010), it is tempting to speculate that brain-derived estradiol levels would also be decreased in the brain after surgical loss of ovarian hormones. It has been reported that the inhibition or null mutation of brain aromatase results in accelerated neurodegeneration (Azcoitia et al., 2003). Furthermore, genetic variants in human aromatase have been reported to confer an increased risk for AD (Iivonen et al., 2004; Huang and Poduslo, 2006). Depletion of aromatase in an animal model of AD also led to earlier and more severe neuropathology than what was observed in OVX control mice, suggesting that depletion of brain-derived estrogen rather that peripheral blood estrogen is a more direct and significant risk factor for developing AD and points to the importance of preserving neurosteroidogenesis for healthy brain aging (Cui et al., 2013). Indeed, targeting key enzymes involved in brain estrogen production have been proposed as pharmacological targets to ameliorate brain function decline during aging and to prevent neurodegenerative diseases (Veiga et al., 2004).

### Sex Hormones and Neuroinflammation during Aging

As reviewed so far, a wide variety of profound physiological changes occur during aging in the brain. The immune system is not an exception, shifting from a resting, surveying state to a chronic mild inflammatory one (Nissen, 2017). Neuroinflammation is choreographed by microglia and astroglia, both of which are affected with aging. Microglia constitute the resident immunocompetent cells of the CNS. It modulates the inflammatory response under pathological conditions but it also maintains homeostasis in the healthy brain through immune surveillance of the brain parenchyma. Changes in microglial cells during aging and in neurodegenerative processes as well as sex-related differences have recently been reviewed (von Bernhardi et al., 2015; Nissen, 2017). Aging promotes the dysregulation of microglia. As a result, an impairment of their physiological neuroprotective functions occurs. At the same time, a mild chronic inflammatory environment characterized by an increased production of inflammatory cytokines and ROS takes place in the CNS. Aged microglia displays morphological changes and a less dynamic response to injury; however, they appear to be activated under mild stimulatory events or minor injuries, responding in an exacerbated way to local and peripheral signals (Nissen, 2017).

Sex differences in gene expression across age in adult human brain have been reported. Thus, women display higher age-related increases in expression of genes associated with immune and inflammatory functions than men (Christensen and Pike, 2015). While both men and women show increased expression of these genes in the hippocampus and entorhinal cortex, only women have significant increases in other brain regions, suggesting a more global pro-inflammatory state in the aged female brain (Berchtold et al., 2008). Also, postmenopausal women display higher expression of macrophage-associated genes in the aging frontal cortex than premenopausal women, suggesting that ovarian hormone loss shifts the microglia phenotype from the resting towards the reactive state (Sárvári et al., 2012).

It is well known that activated microglia can be polarized into a proinflammatory/cytotoxic M1 or an anti-inflammatory/neuroprotective M2 phenotype in response to a myriad of physiological and pathological stimuli (Villa et al., 2016; Labandeira-Garcia et al., 2017). It has been broadly reported that estrogens trigger the polarization of microglia to an M2 phenotype (Habib and Beyer, 2015). This estrogen action becomes especially relevant under chronic inflammation conditions, where perpetuation of microglial proinflammatory status induce neuronal damage. Hence, it could provide an explanation for the neuroprotective effects of estrogens observed in aging and neurodegenerative diseases (Gyenes et al., 2010; Selvamani et al., 2012; Siani et al., 2017). Besides, growing evidence has highlighted the role of the local renin-angiotensin system in aging and several processes mediated by microglial activation and neuroinflammation (Hellner et al., 2005; Kerr et al., 2005; Rey et al., 2007; Rodriguez-Pallares et al., 2008; Torika et al., 2016). Interestingly, estrogen-induced inhibition of this system leads to reduced oxidative stress, neuroinflammation, and neurodegeneration of dopaminergic neurons in murine models of PD, which may explain, at least in part, the lower risk of developing the disease in premenopausal vs. postmenopausal women and men (Rodriguez-Perez et al., 2010; Labandeira-Garcia et al., 2016).

Like humans, female rodents display enhanced inflammation in the brain with aging, which is regulated, at least in part, by estrogen status. When compared to age-matched males, female mice show higher induction of inflammation-related genes, especially of microglia-specific ones, in the hippocampus (Mangold et al., 2017). In addition, gene expression studies in the rat frontal cortex and hippocampus have shown that both aging and ovarian hormone loss increase the expression of several microglial and immune function genes, leading to a shift towards a more inflammatory and reactive microglia phenotype (Sárvári et al., 2011, 2012, 2014). Estrogen replacement treatment attenuates the OVX-induced effects and most of these effects are mimicked by both ERα and ERβ agonists, suggesting that both ERs are targets of estrogens in microglia (Sárvári et al., 2011, 2014). Besides, both aging and OVX increase the gene expression of pro-inflammatory cytokines such as TNFα and IL-1β in the hippocampus of aged mice (Benedusi et al., 2012). It has been shown that aging exacerbates microglial response to OVX, indicating that loss of sex hormones increase the susceptibility of aged microglia to inflammation (Lei et al., 2003). Recently, it has been suggested that a form of estrogen resistance may be involved in the impaired ability of microglia to resolve inflammation during aging (Villa et al., 2016). Astroglial cells also display a proinflammatory phenotype during aging, expressing and secreting increased levels of inflammatory markers such as TNF-α, IL-1β and IL-6 and hence contributing to brain neuroinflammation. They also display age-related increased levels of intermediate glial fibrillary acidic protein (GFAP) and vimentin filaments as well as increased accumulation of proteotoxic aggregates (Salminen et al., 2011). Taken together, both preclinical and clinical data indicate that both aging and menopause lead to increased neuroinflammation, which may contribute to sex differences in age-related neurological diseases such as stroke and AD.

Although the age-induced increase in the expression of GFAP has been the most classical change reported in astroglial cells (Schipper, 1996; Unger, 1998; Cotrina and Nedergaard, 2002; Lynch et al., 2010), experimental data suggest that physiological brain aging and early stages of neurodegenerative disease are characterized by an increased number of dystrophic astrocytes (Oddo et al., 2003; Broe et al., 2004; Mena and García de Yébenes, 2008; Rossi et al., 2008; Bradford et al., 2010; Olabarria et al., 2011; Cerbai et al., 2012; Kulijewicz-Nawrot et al., 2012; Beauquis et al., 2013). These cells are smaller and less complex, with reduced capacity for glutamate uptake and decreased activity of glutamine synthetase, hence displaying reduced neuroprotective and homeostatic potential (Verkhratsky et al., 2014).

**Figure 1:**
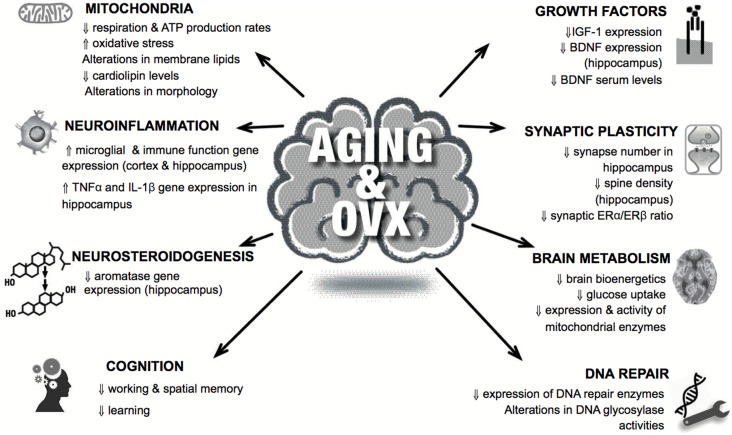
Organismal, cellular and molecular hallmarks of brain aging are frequently observed in ovariectomized animal brains. Examples of common features shared in aging and lack of sex hormones in the brain are shown. For details, see text.

Taken together, aging promotes profound changes in morphological and functional parameters within the brain, most of which are recapitulated by sex hormone loss, particularly in the female (Figure 1).

## DNA Repair and Brain Aging

The DNA in brain cells is frequently damaged and if this damage is not removed it can have serious consequences such as compromised genomic stability. Although exogenous sources of DNA damage exist and replication errors may lead to DNA strand breaks, the majority of DNA lesions in non-replicating brain cells are introduced endogenously by ROS. It is important to note, though, that there are other common DNA lesions that have recently been recognized to also substantially impact genome stability. Among these, rNTP incorporation into DNA, genome damage from transcription-associated R-loop formation (hybridization of nascent primary RNA transcripts to the transcribed DNA strand), and aberrant topoisomerase activity are potential threats to the neural genome (McKinnon, 2016; Williams et al., 2016). Also, the generation of DNA damage through topoisomerase I cleavage complexes formed during transcription is likely to play a significant role in neurons due to the high transcription rates in these cells (Katyal et al., 2014). The difference in replicative status influences to some extent the damage and repair processes in cells. In this context it is important to note that the brain is composed of both non-dividing and dividing cells. As mentioned above, neurons are in a post-mitotic state. But glial cells (e.g., astrocytes, oligodendrocytes and microglia) are in either a proliferative or non-proliferative state, depending on their differentiation status. Because both neurons and glial cells are required to carry out the various higher-order brain functions, it is critically important to maintain all cell types in an appropriate number and configuration (Iyama and Wilson, 2013).

When DNA is exposed to ROS, it may result in oxidative base modifications. Among these, 8-oxo-dG is one of the most abundant and well characterized (Dizdaroglu et al., 2002). It has been estimated that approximately 180 guanines are oxidized to 8-oxo-dG per mammalian genome per day (Lindahl, 1993). These lesions may cause G:C to T:A transversion mutations because 8-oxo-dG can base pair with adenine as well as cytosine during DNA replication (Shibutani et al., 1991). 8-oxodG has also been implicated in an event called transcriptional mutagenesis (TM), whereby a mis-incorporated adenine in the transcribing mRNA leads to the generation of mutated species of protein (Bregeon et al., 2009). Interestingly, it has recently been suggested that TM may contribute to α-synuclein aggregation and the pathogenesis of PD (Basu et al., 2015). It is important to note that e.g., 8-oxo-dG may also become further oxidized into secondary oxidation products such as guanidinohydantoin 2′-deoxynucleoside (dGh) in doublestranded DNA or spiroiminodihydantoin 2′-deoxynucleoside (dSp) in single stranded DNA (Suzuki et al., 2001; Fleming and Burrows, 2013). ROS may also give rise to DNA single strand breaks (SSBs), which are some of the most common DNA lesions arising at an estimated rate of tens of thousands per cell per day (Lindahl, 1993). Persistent SSBs can lead to the collapse of the replication fork during chromosome duplication, but they may also block transcription. Double strand breaks (DSBs) may also be formed as a result of the actions of ROS. Although these lesions normally are relatively rare, DSBs are some of the most deleterious forms of DNA damage, causing translocations and loss of genomic information. Finally, ROS can also lead to lipid peroxidation, whose byproducts can also react with DNA to produce exocyclic DNA lesions (Yu et al., 2016).

There is growing evidence for the accumulation of unrepaired DNA lesions in the CNS during both normal and accelerated aging and progressive neurodegeneration, but the observed changes depend on brain region, cell types and sub-cellular location of the DNA. Rutten et al. (2007) reported that the number of SSBs increases substantially with aging in the nuclear DNA (nDNA) of hippocampal pyramidal and granule cells as well as in cerebellar granule cells but not in cerebellar Purkinje cells in the mouse brain. For rat brain it has been reported that Ogg1-sensitive sites (i.e., mainly 8-oxo-d-G) accumulate continuously through adulthood and old age in both neurons and astrocytes (Swain and Subba Rao, 2011). Furthermore, it has been shown that susceptibility to oxidative DNA damage is lower and BER capacity is higher in undifferentiated human SH-SY5Y neuro-blastoma cells than in neuronally differentiated SH-SY5Y cells (Sykora et al., 2013). Due to the close proximity of the mtDNA to the site where most of the cellular ROS is formed, mtDNA is particularly vulnerable to oxidative damage. Accordingly, age-associated accumulation of DNA damage is mostly reported for mtDNA. It has been reported that human brain cells experience a progressive increase in the levels of 8-oxo-dG and the magnitude of the age-related damage is approximately ten-fold greater in mtDNA than in nDNA (Wang et al., 2005, 2006; Lovell and Markesbery, 2007).

**Figure 2:**
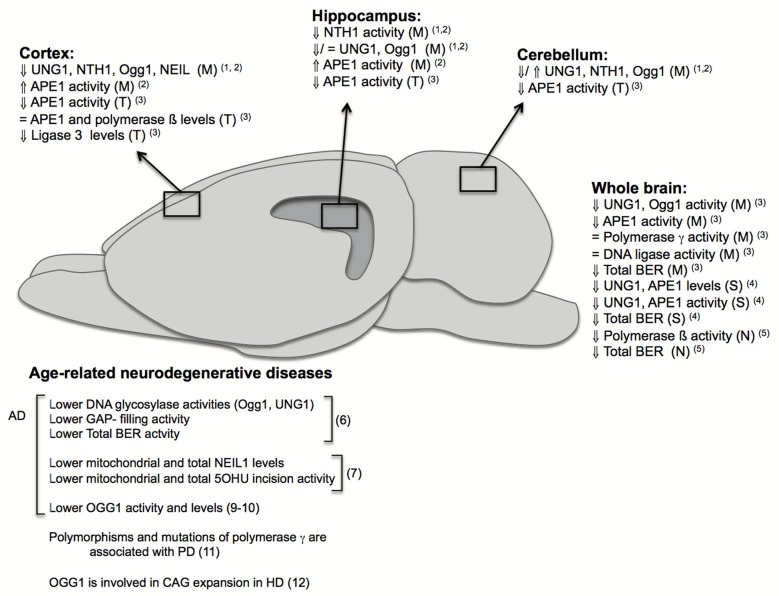
Changes in base excision repair (BER) in brain aging and neurodegeneration. The figure depicts age-related changes in the BER pathway from different investigations performed in specific brain regions: cortex, hippocampus, cerebellum; or using whole brains and in different fractions: (M): mitochondrial, (N): nuclear, (S): synaptosomal, (T): total. It also summarizes some of the major findings in humans and rodents implicating BER in age-related neurodegenerative diseases. Numbers in brackets correspond to references: (1) Imam et al. (2006); (2) Gredilla et al. (2010b); (3) Kisby et al. (2010); (4) Gredilla et al. (2012); (5) Cabelof et al. (2003); (6) Weissman et al. (2007b); (7) Canugovi et al. (2014); (8) Lovell et al. (2000); (9) Iida et al. (2002); (10) Shao et al. (2008); (11) Coppedè and Migliore (2015); (12) Kovtun et al. (2007).

If left unrepaired, DNA damage can give rise to genomic instability and trigger signaling cascades leading to cellular senescence or cell death, which are phenotypes associated with aging (Rodier et al., 2009). Accordingly, increased levels of mutations in the DNA have been described to occur in the brain and other tissues during normal aging leading to DNA instability (Gredilla, 2010). Although the majority of known DNA repair pathways are present in neurons and glia cells, many investigations suggest that BER impairment and increased DNA instability are the principal contributors to brain aging and age- associated neurodegenerative diseases (Figure 2). The BER pathway is basically divided in four distinct steps. First, DNA glycosylases recognize and remove the modified bases. These DNA glycosylases, such as NTH1 and Ogg1, have distinct substrate specificities. They render an abasic site, which is mainly processed by the AP endonuclease (APE1). BER may proceed through two different sub-pathways, both in nuclei and mitochondria: short- or long-patch BER. Both pathways mainly differ in the number of nucleotides that are incorporated into the gap by a DNA polymerase. Different accessory proteins are involved in this step. Finally ligation of the DNA strand takes place by a DNA ligase (Robertson et al., 2009; Liu and Demple, 2010).

Together with BER, it has been suggested that proteins involved in MMR and DSBs repair are present in mitochondria; however, no evidence of mitochondrial NER activity has been reported (Gredilla et al., 2010a). The NER pathway repairs different types of helix distorting and bulky lesions. Moreover, this pathway plays a critical role in DNA crosslinks repair. Similarly to other DNA repair pathways, NER involves various steps, including damage recognition, opening of the DNA helix, incision of the nucleotides surrounding the lesion, gap filling and ligation. Two NER sub-pathways exist: the global genome (GG)-NER and transcription coupled (TC)-NER. The first step of the pathway, recognition of the damage, involves the action of multiple proteins. In GG-NER destabilization of the base pairing is detected by XPC together with the human homolog Rad23 protein, which is suggested by many studies to be the first protein factor to arrive at the lesion. For specific types of lesions, such as UV-induced photoproducts, other proteins are involved i.e., UV-damaged DNA-binding protein (UV-DDB), thereby recruiting XPC and extending the substrate specificity (Sugasawa, 2011). In TC-NER, damage recognition is caused by the blockage of the transcribing RNA polymerase II on the damaged DNA template. TC-NER is initiated by the CSB protein, followed by CSA. In both GG-NER and TC-NER, the lesion recognition step is followed by recruitment of TFIIH. DNA is unwound around the lesion and the open complex is stabilized by XPG and XPA. Specific endonucleases XPG and ERCC1/XPF cleave the lesion, and after removal of a 24–32 nucleotide fragment, the remaining single-strand gap is filled by the replication machinery and the resulting nick is sealed by ligase I or ligase III (de Boer and Hoeijmakers, 2000; Jeppesen et al., 2011). In MMR, MSH2–MSH6 or MSH2–MSH3 heterodimeric ATPase complexes recognize and bind the mismatch. After recruitment of different proteins e.g., MLH1, PMS2, PCNA, excision is performed by the exonuclease EXO1. Repair synthesis is accurately performed by Polδ, and ligation of the remaining nicks after DNA synthesis is performed by ligase I (Jiricny, 2006). The DSB repair pathway is regulated by several phosphorylation events, starting immediately after DSB formation, where large numbers of the histone protein H2AX are phosphorylated (γH2AX) and accumulate in the chromatin around the break (Bonner et al., 2008; Muslimovic et al., 2008). Moreover, different DSB damage response proteins accumulate in foci around DSBs, activating signaling pathways that affect events related to DNA repair, cell cycle checkpoints, and transcription. The repair of DSBs involves one of two mechanisms: non-homologous end-joining (NHEJ), directly joining the broken ends and involving loss of genetic material, or homologous recombination (HR), which can only take place in replicating cells and uses the intact sister chromatid as a template for repair.

Several studies have shown that oxidative DNA damage do accumulate with age, especially in the mitochondria. The DNA glycosylase Ogg1, is likely to be particularly important for DNA maintenance in the brain due to its specificity for the common 8-oxo-dG lesion. Ogg1 activity decreases significantly with age in neuronal extracts of rat brain, and a similar trend is observed, to a lesser extent though, in rat brain astrocytes (Swain and Subba Rao, 2011). The glycosylases NEIL1 and NEIL2, are also considered to be essential for neuronal DNA repair, due to their ability to remove various oxidatively damaged bases in single stranded regions of DNA, since neuronal DNA is heavily transcribed and during that process the DNA is transiently in a single stranded conformation. In aged rats the activity of APE1, the major enzyme responsible for incision of the DNA backbone in BER, is reduced in the frontal/parietal cortex, cerebellum, brainstem, midbrain and hypothalamus compared to young rats (Kisby et al., 2010) and APE1 activity seems to be reduced both in neurons and astrocytes (Swain and Subba Rao, 2011). While APE1 activity declines with age, there does not seem to be any change in the protein levels of either APE1, Pol β or LIG3 in the rat frontal/parietal cortex, suggesting that the reduced APE activity could be due to altered post-translational modification.

Mitochondrial DNA repair is likely to be particularly important due to the heavy exposure of mtDNA to ROS. Studies by Hollensworth et al. (2000) indicate that mtBER is more efficient in astrocytes than oligodendrocytes or microglia, and that the efficient repair associates with reduced susceptibility to apoptosis. We have previously reported age-associated changes in mtBER activity in the murine brain and showed that these changes are region specific. Thus, in cortical mitochondria, DNA glycosylase activities peak at middle-age followed by a significant drop at old age. However, only minor changes are observed in hippocampal mitochondria during the whole lifespan. Furthermore, DNA glycosylase activities are lower in hippocampal than in cortical mitochondria (Gredilla et al., 2010b). Noteworthy, we have also reported that an age-related decline in mouse brain mtBER occurs specifically at the synapses, which is associated with a decrease in the level of BER proteins (Gredilla et al., 2012). In the human brain, the expression of at least some genes coding for proteins involved in BER has been shown to fluctuate with aging. Interestingly, this seems to some extent to be due to an age-associated accumulation of oxidative lesions in the promotor regions of these genes (Lu et al., 2004). Recently, work from Lillenes et al. (2011, 2017), suggests a potential link between specific SNPs in APE1 and DNA polymerase β in humans and reduced cognitive performance in healthy elderly individuals, which is in support of a role for BER in the maintenance of brain function late in life.

## Sex Hormones and DNA Repair in the Brain

As mentioned above, DNA instability is one of the major hallmarks of aging. Several investigations have associated brain aging and age-related neurodegenerative disorders with higher accumulation of DNA mutations due, at least in part, to a reduction in DNA repair capacity (Vermulst et al., 2007; Jeppesen et al., 2011; Sanders et al., 2014). Since sex steroids have been described to exert neuroprotective effects, it is likely that such effects might be partly linked to a direct impact on DNA repair mechanisms. In fact, the effect of sex steroids on DNA damage responses have been extensively investigated in cancer, where sex steroids have been described to interact with different DNA repair pathways (Caldon, 2014). Estrogen and androgens positively regulate the repair of DSBs by activation of NHEJ in breast and prostate cancers (Schiewer and Knudsen, 2016). However, conflicting results have been reported regarding the effect of sex steroids on HR depending on the type of cancer, with positive regulation in prostatic cancer and melanoma (Fang et al., 2013; Bowen et al., 2015), but negative in medulloblastoma (Urbanska et al., 2009). Estrogen has also been associated with an enhanced DNA repair via MMR in colorectal cancer (Lu J. Y. et al., 2017). Regarding NER, estrogen up-regulates the repair of UV-induced DNA damage in breast cancer cells (Boulay and Perdiz, 2005) while reducing the repair of thymine dimers in human keratinocytes (Evans et al., 2003). Moreover, in patients with basal cell carcinoma, postmenopausal women show a significant drop in lymphocyte DNA repair capacity compared to postmenopausal women on estrogen supplementation (Grossman and Wei, 1995).

Poly (ADP-ribose) polymerases (PARP) are members of a family of enzymes that are particularly abundant in cell nuclei and can function as sensors of DNA damage. Various investigations have reported sex differences in PARP1 activity. A recent report has shown that female and estrogen-treated male mice are completely protected from alkylation-induced nephrotoxicity in a transgenic model of enhanced alkyladenine DNA glycosylase (Aag) expression together with PARP-1 KO (AagTg/Parp1−/−). This sex dimorphism suggests a direct interaction between Aag and/or PARP1 with estrogen pathways leading to changes in DNA repair activity and/or gene expression (Calvo et al., 2016). Estrogen supplementation also affects PARP-1 activity differently in peripheral blood mononuclear cells from male and female mice (Zaremba et al., 2011). Moreover, PARP-1 is an important regulator of neuronal cell death and cellular responses to DNA damage and it has been reported that PARP-1 mediated cell death is dependent on androgen-receptor signaling after stroke (Vagnerova et al., 2010).

In contrast to cancer research, the relation of sex steroid hormones and DNA repair pathways and whether the former regulates the latter contributing to their neuroprotective effect has not been extensively investigated. However, various studies have reported that sex steroids, particularly estrogen and progesterone, regulate DNA repair mechanisms in the brain. Most of those studies have used OVX animals as a model for analyzing their effect on specific activities or expression of DNA repair enzymes. Studies on how estrogen levels affect DNA repair in the brain have mainly focused on enzymes involved in BER. Estrogen has been described to regulate the transcription as well as the translocation within different cellular compartments of BER enzymes. Neuroprotection of estrogens has been shown in cerebral cortex where they have been described to reduce oxidative DNA damage after hypoxia (Rao et al., 2011), an effect that is associated with an enhancement in the transcription of DNA repair enzymes like APE1 in that brain region (Dietrich et al., 2013). Moreover, estrogen supplementation in OVX old female macaques has been shown to increase the transcription levels of different DNA repair enzymes in the dorsal raphe (Bethea et al., 2016). Bethea et al. (2016) described a significant increase in the transcription of DNA repair enzymes involved in different pathways, including BER (APE1, NTH1 among others), NER (RAD23 and GTF2H5, a subunit of THIIF) and HR (NBS1 and SHFM1). Interestingly, they also reported that when estrogen is combined with progesterone the effect is reduced or even absent (Bethea et al., 2016). That is in agreement with those studies that have previously reported an antagonistic effect of progesterone over estrogen, e.g., in cognitive function of hormone-treated OVX rats (Bimonte-Nelson et al., 2006) and in hippocampal cellular survival after treatment with kainate or mitochondrial toxins (Rosario et al., 2006; Carroll et al., 2008; Yao et al., 2011). Similarly, progesterone has also been described to reduce the estrogen-related enhancement of BDNF in OVX rats (Bimonte-Nelson et al., 2004). This is especially interesting, because BDNF has been described to enhance neuronal survival, at least in part by inducing the transcription of DNA repair enzymes such as APE1 (Yang et al., 2014). Thus, the effect of estrogen on DNA repair might also be related to BDNF levels, since as we previously described, estrogens induce BDNF expression. Various studies have also linked beneficial effects of estrogens with an up-regulation of Nrf-2 via the PI3K/Akt signaling pathway. This is a relevant link since Nrf2 has been associated to the transcription of antioxidant response elements, including different DNA repair enzymes (Jayakumar et al., 2015; Habib et al., 2016). The expression of Nrf2 has been described to be reduced after OVX and restored to normal levels after estrogen supplementation in murine hippocampus (Li et al., 2017). This mechanism has also been described to play an important role in the protective role of estrogens after light-induced degeneration in retina (Zhu et al., 2015). Moreover, it has been described that certain phytestrogens exert their beneficial effects by activating the PI3K/Akt/Nrf2 pathway through ER binding (Hwang and Jeong, 2010).

Estrogen not only regulates the expression/activity of DNA repair enzymes, it has also been described to regulate their subcellular distribution. Leclère et al. (2013) have shown that in whole brain extracts, APE1 activity is increased in mitochondrial fractions after OVX. The same effect is observed in liver extracts, being associated with a translocation of APE1 from the cytosol to the mitochondria. Moreover, this effect of estrogen on the trafficking of DNA repair enzymes has been suggested to be brain region-dependent. Araneda et al. (2005) have shown that after estrogen supplementation in OVX rats, Ogg1 was translocated within the nucleus and to other cellular compartments in the paraventricular nucleus of the hypothalamus but not in the bed nucleus of the stria terminalis. This translocation of DNA repair enzymes dependency on estrogen levels might be associated with the increased oxidative stress that has been described to occur after OVX (Borrás et al., 2003; Baeza et al., 2008). Various studies have reported that DNA repair enzymes can be specifically translocated to nuclei and mitochondria in response to increased oxidative stress and DNA damage (Mitra et al., 2007; Boesch et al., 2011; Li M. X. et al., 2012).

## Concluding Remarks

Brain aging is associated with an important decline in neuronal function. The drop in sex hormone levels during aging is believed to play an important role in the loss of neuronal function, which may further contribute to the onset of age-related neurodegenerative diseases. A broadly used animal model for investigating the effects of sex hormones in brain aging and neurodegeneration is gonadectomy, especially in rodents. Gonadectomized young rodents display several features of intact aged animals, including changes in brain metabolism, mitochondrial function, and neuroinflammation among others. Interestingly, estrogen supplementation in female rodents has been described to revert the negative effects of OVX in brain functionality. Different studies have supported the neuroprotective effect of estrogen at different levels. Such effect may be extremely complex, and it may depend not only on the type of receptor involved, but also on the timing of estrogen therapy. In view of ERβ sustained actions on plasticity during aging in the female brain and its effects on mitochondrial function, ERβ appears as a target worth exploring to counteract age-associated detrimental effects in the brain. A better understanding of the underlying mechanisms of sex hormone actions may lead to new avenues for treatment of age-associated neurodegeneration. Recently, some studies have reported a significant effect of estrogens on DNA repair enzymes in the brain. However, the investigations on this particular issue and the related mechanisms are still scarce. Since reduction in DNA repair capacity has been suggested to contribute to brain aging and the onset of neurodegenerative diseases, it is critical to fully understand how estrogens affect DNA repair mechanisms. This novel interplay warrants further study in an attempt to find new therapeutic targets to promote healthy brain aging and prevent age-related diseases.

## Author Contributions

All authors listed, have made substantial, direct and intellectual contribution to the work, and approved it for publication.

## Conflict of Interest Statement

The authors declare that the research was conducted in the absence of any commercial or financial relationships that could be construed as a potential conflict of interest.
